# A Rare Case of an Intrasellar Arachnoid Cyst With Middle Cranial Fossa Extension

**DOI:** 10.7759/cureus.41022

**Published:** 2023-06-27

**Authors:** Joseph Silva, Giulia X Bornancin, Ricardo Ramina

**Affiliations:** 1 Neurosurgery, Neurological Institute of Curitiba, Curitiba, BRA; 2 Neurological Surgery, Neurological Institute of Curitiba, Curitiba, BRA; 3 Neurological Surgery, Instituto Neurológico de Curitiba, Curitiba, BRA

**Keywords:** middle fossa cyst, case report, arachnoid cyst, sellar cysts, sellar arachnoid cyst

## Abstract

Intrasellar arachnoid cysts represent around 1% of all selar lesions. Generally, patients are asymptomatic and when they exhibit visual and/or hormonal disturbances, the indication for surgery is prompted. A 51-year-old woman with a known purely intrasellar arachnoid cyst diagnosed 23 years prior to presentation, evolved with gradual campimetric evaluation. Magnetic resonance imaging showed significant growth of the lesion, now extending into the left middle fossa through the cavernous sinus. The patient underwent cyst fenestration via the transsphenoidal approach. This is the first case in the literature of a patient with an intrasellar arachnoid cyst extending into the middle cranial fossa.

## Introduction

Intrasellar arachnoid cysts represent around 1% of all sellar lesions and only three percent of arachnoid cysts, they are rarely seen in this localization with few reports available in the literature [1]. It enters into the differential diagnosis of adenomas, craniopharyngiomas, and Rathke’s cleft cyst. The most common location of arachnoid cysts is in the Sylvian fissure followed by the cerebellopontine angle. Based on the variety of possible diagnoses and the rarity of this type of lesion, as well as the difficulty in making a therapeutic decision, we describe a case of a sellar arachnoid cyst extending into the middle cranial fossa. Sellar arachnoid cysts with parasellar extension have not been previously described [1].

## Case presentation

**Figure 1:**
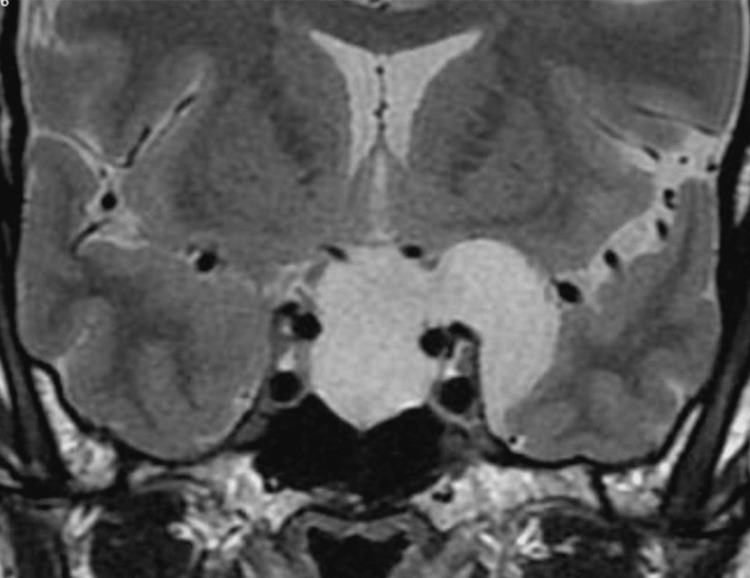
Coronal T2- weighted magnetic resonance imaging of the sellar region, showing hyperintense cavity within the sella, extending into the left middle fossa through the superior part of the cavernous sinus

A 51-year-old woman, with a known purely intrasellar arachnoid cyst, was diagnosed 23 years ago after a headache episode, remaining asymptomatic after the diagnosis, she had no medical history. She continued to follow-up with image surveillance, showing a stable lesion until 2019, magnetic resonance imaging (MRI) evidenced growth of the cyst, with extension to the suprasellar cistern and left middle fossa (Figure 1) and surgery was indicated.

Campimetric evaluation showed left temporal hemianopia and right superior temporal quadrantanopia so surgical treatment was proposed. Preoperative pituitary hormones were within normal range.

**Figure 2:**
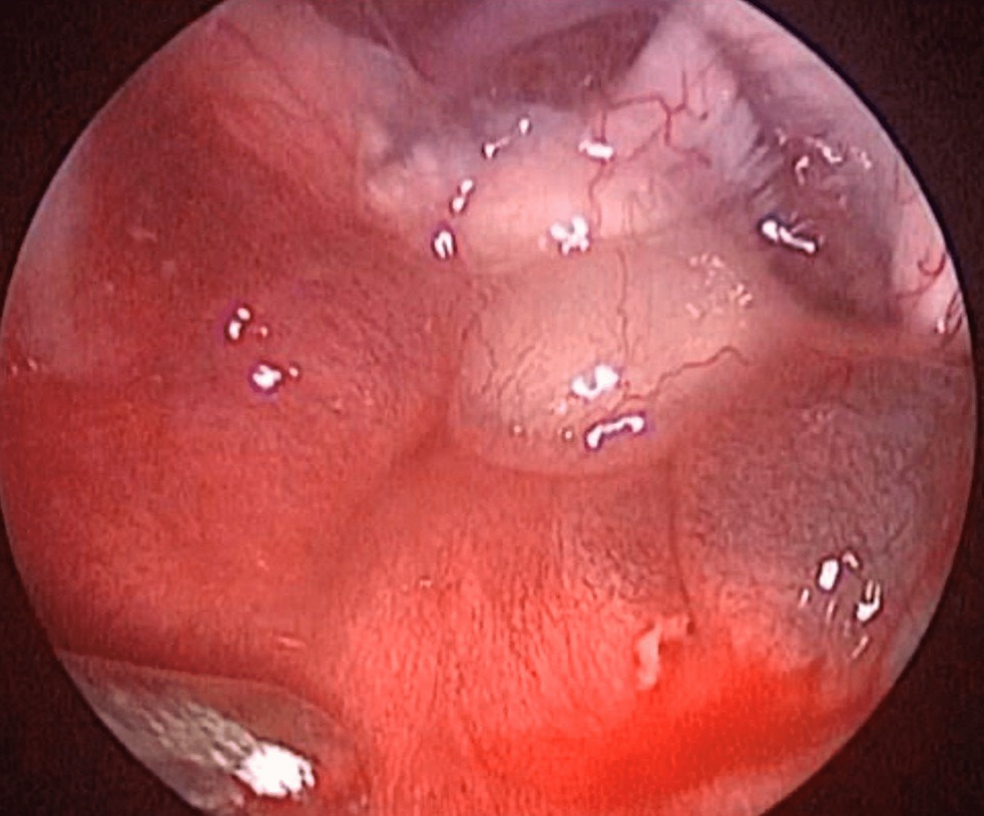
Intraoperative aspect through the endoscopic transsphenoidal approach showing the thinned floor of the sella

A right mono-nostril endoscopic transsphenoidal approach was performed. After the opening of the sphenoidal sinus, it was noticed that the floor of the sella was thinned (Figure 2).

**Figure 3:**
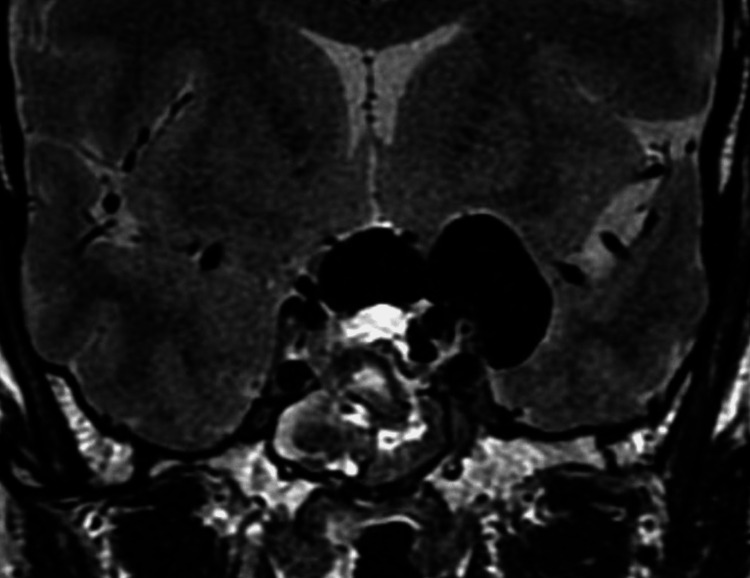
Postoperative coronal T2-weighted magnetic resonance imaging of the sellar region, showing air inside the cyst, as well as a significant reduction of the size of the cyst

After the opening of the sella and dura, the cyst was drained. Direct endoscopic visualization of the cavity showed the pituitary gland pushed back against the dorsum sellae, and the optic chiasm and optic nerves pushed upward with evidence of atrophy. Fenestration of the cyst to the interpeduncular cistern was then performed to avoid recurrence. Closure of the cavity was achieved with oxidized cellulose, fibrin glue, fat, and fascia obtained from the abdominal wall, and nasal cartilage, followed by repositioning of the nasoseptal flap, sterile compressed sponge, and nasal packing. A lumbar drain was left in place for 72 hours to reduce the chance of a CSF leak. Postoperative MRI showed complete drainage of the cyst (Figure 3).

The early postoperative period was uneventful and the patient was discharged on the fourth postoperative day. In his neurosurgical follow-up of one year, no abnormality was observed.

## Discussion

The arachnoid cyst was first described in Bright’s Reports of Medical Cases in 1831 [1]. Up to 2016, less than 100 cases of intrasellar cysts have been published in the English literature, corresponding to 1% of intrasellar lesions. The main hypothesis for arachnoid cyst formation is due to congenital membrane duplication during embryogenesis, which Meyer et al. believe is also the cause of intrasellar cysts [2], whereas other authors propose a defect of the sellar diaphragm that allows the arachnoid membrane to insinuate [3-7].

Generally, a sellar arachnoid cyst is an asymptomatic lesion, an incidental finding in brain MRI, and about 50% are found in the Sylvian fissure [6,8]. Sellar arachnoid cysts usually affect older patients and when it produces symptoms, these are similar to other pituitary lesions - visual deficits, headache, and hormonal disturbances, which prompt surgical intervention [4,6,9].

When surgery is proposed for a sellar arachnoid cyst, it usually is done by a transsphenoidal or transcranial approach [6]^.^ Regardless of the approach, patients usually have a good recovery with progressive improvement of the preoperative deficits [10]. The main complication arising from a transsphenoidal approach for arachnoid cyst is a cerebrospinal spinal fluid leak though recent reports have stated its safety in these cases [8].

## Conclusions

A parasellar extension of a sellar arachnoid cyst has not been previously reported. These are rare lesions that seldom require treatment, unless they become symptomatic. Deciding which is the safest access route and its possible complications makes the best treatment possible. The endocopic transsphenoidal approach is a safe way to access these lesions with good patient outcomes.
